# REDCap as a Platform for Cutaneous Disease Management in Street Medicine: Descriptive Study

**DOI:** 10.2196/48940

**Published:** 2024-01-09

**Authors:** Emily Eachus, Kayla Schwartz, Taha Rasul, Daniel Bergholz, Jonette Keri, Armen Henderson

**Affiliations:** 1 Miami Street Medicine University of Miami Miller School of Medicine Miami, FL United States

**Keywords:** REDCap, unsheltered homelessness, street medicine, informatics, cutaneous, homeless, homelessness, data capture, data collection, skin, dermatology, vulnerable, low income, low resource, database, chart, health record, health records, EHR, electronic health record

## Introduction

According to the 2022 Annual Homelessness Report to Congress, on a single night, 582,462 people experienced homelessness across the United States, and 233,832 (over 40%) of those experienced unsheltered homelessness [1]. A 2020 systematic integrative review of health and social care in people experiencing homelessness showed that this population experienced inequities in access to basic human needs, health care, and social support [2], which are compounded by poor interpersonal dimensions such as a lack of provider support and stigmatization. Altogether, people experiencing homelessness are at risk for morbidity and premature death [3,4]. People experiencing homelessness require programs that bypass social barriers to health care. The street medicine approach uses teams of health care providers and volunteers to meet patients where they are currently living on the streets of major cities, bypassing barriers such as lack of transportation, ability to pay, and lack of primary care by bringing a mobile clinic with medications, supplies, and providers directly to people experiencing homelessness [5].

Because student-led street medicine is often volunteer based and not directly affiliated with hospital systems, many lack robust electronic medical record (EMR) systems [6]. Correspondingly, the lack of efficient medical care documentation is an obstacle to providing longitudinal care to patients experiencing homelessness. REDCap is a Health Insurance Portability and Accountability Act–compliant free web application used to create databases for clinical research and projects [7,8]. However, per our evaluation of the medical literature, there are no reports of medical record keeping or using REDCap among street medicine organizations.

This retrospective descriptive study describes the use of a custom REDCap-based EMR for the management of cutaneous diseases in a Miami-based street medicine organization, Miami Street Medicine (MSM).

## Methods

### Ethical Considerations

The University of Miami Institutional Review Board (IRB) approved reviewing records of cutaneous disease among people experiencing homelessness (IRB ID: 20230666).

### Overview

A custom REDCap-based EMR was developed in November 2020 for MSM. The MSM custom REDCap includes forms for medical notes, vitals, labs, and more. The EMR was further customized to the unique needs and circumstances of people experiencing homelessness.

Specific drop-down lists about cutaneous pathology were created. The drop-down menus allow for selecting a location on the body, wound characterization, whether the wound was infected, if debridement was done, and supplies used.

Between July 2021 and January 2022, patients were seen curbside in Miami once per week. Patients were assigned medical record numbers and had medical histories taken, vitals examined, and medications distributed as needed or called into a pharmacy by an attending physician. Records about skin and nail complaints were reviewed by board-certified dermatologists who made diagnoses of cutaneous conditions, recommended medical plans, and called in prescriptions. Diagnoses were not based on standard codes, but rather on clinical expertise, as all services were free and not reported to health insurance agencies.

Skin and nail pathologies were categorized by diagnosis as chronic infections, acute infections, inflammatory, wounds, miscellaneous, nail disorders, and undetermined.

## Results

Among 140 patients experiencing homelessness seen from July 2021 to January 2022, 112 skin and nail diagnoses were recorded. The sample included a diverse cohort that was 50.2% (n=56) Black and 45.8% (n=51) White, with the remainder being Asian or Native American patients. Hispanic patients of any race made up 34.8% (n=39) of the sample. A total of 68.1% (n=77) of patients identified as male and 31.9% (n=35) as female. The highest morbidity lesions resulting in disability or infection were chronic wounds and ulcers requiring multiple care instances.

The most common dermatologic diagnosis outside of the miscellaneous category was acute infections, with the most common type of medication dispensed being for wound care (Multimedia Appendix 1).

## Discussion

The use of a free customizable REDCap EMR system was instrumental in recording the high burden of cutaneous diseases and connecting patients with specialists and follow-up care. Charitable health care organizations can use REDCap as it provides cost-effective, modifiable, and accessible management of patient data. One of the benefits of using REDCap as an EMR for special populations is its customizability and ease of data analysis.

Limitations of using the REDCap EMR include data entry errors by volunteer scribes and the great effort required to build and maintain this system. As a transient population, we noted 71.6% (80/112) patient attrition from care. This could be improved by communication via phone or email. Further, many topical medications offered to patients were distributed without documentation; only medications specifically ordered for patients were included in this synthesis.

A REDCap-based EMR is a valuable tool for established street medicine teams and may improve the delivery of care to people experiencing homelessness.

## Appendix

Multimedia Appendix 1 Distribution of skin diagnosis by type.

## Abbreviations

EMR: electronic medical record
MSM: Miami Street Medicine
