# Supplementary figures and images for: REDCap as a Platform for Cutaneous Disease Management in Street Medicine: Descriptive Study
Source: JMIR Dermatol. 2024 Jan 9;7:e48940. doi: 10.2196/48940 (PMC10806445; doi:10.2196/48940)

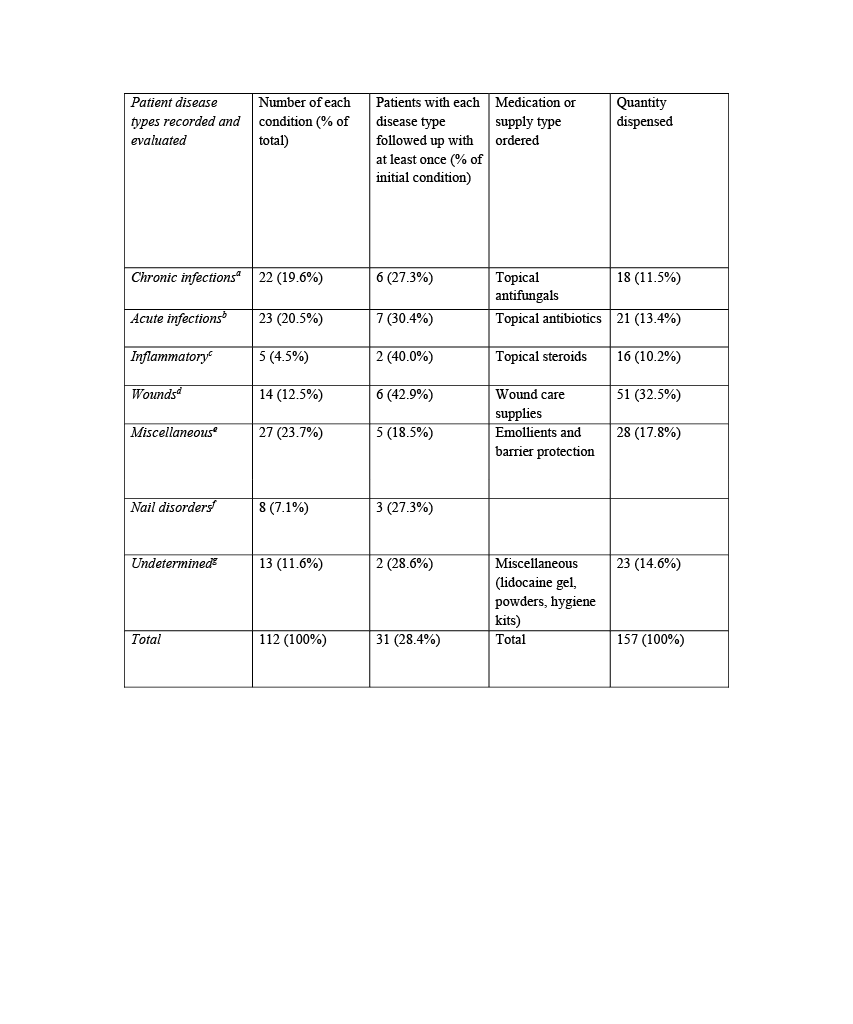

Supplement: Multimedia Appendix 1 [file derma_v7i1e48940_app1.png]
